# Validation of an MRI-only planning workflow for definitive pelvic radiotherapy

**DOI:** 10.1186/s13014-022-02023-4

**Published:** 2022-03-18

**Authors:** Laura M. O’Connor, Jason A. Dowling, Jae Hyuk Choi, Jarad Martin, Helen Warren-Forward, Haylea Richardson, Leah Best, Kate Skehan, Mahesh Kumar, Geetha Govindarajulu, Swetha Sridharan, Peter B. Greer

**Affiliations:** 1 Department of Radiation Oncology, Calvary Mater Hospital, Cnr Edith & Platt St, Waratah, , Newcastle, NSW 2298 Australia; 2 School of Health Sciences, University of Newcastle, University Drive, Newcastle, NSW 2308 Australia; 3 Australian E-Health Research Centre, Commonwealth Scientific and Industrial Research Organisation (CSIRO), Bowen Bridge Rd, Herston, QLD 4029 Australia; 4 School of Mathematical and Physical Sciences, University of Newcastle, University Drive, Newcastle, NSW 2308 Australia; 5 Department of Radiology, Calvary Mater Hospital, Edith Street, Waratah, Newcastle, NSW 2298 Australia

**Keywords:** MRI radiotherapy planning, Radiotherapy, Rectum neoplasms, Cervix neoplasms, Endometrium neoplasms, Anal canal neoplasms, Synthetic CT, Computer assisted radiotherapy planning, Image guided radiotherapy, Intensity modulated radiotherapy

## Abstract

**Purpose:**

Previous work on Magnetic Resonance Imaging (MRI) only planning has been applied to limited treatment regions with a focus on male anatomy. This research aimed to validate the use of a hybrid multi-atlas synthetic computed tomography (sCT) generation technique from a MRI, using a female and male atlas, for MRI only radiation therapy treatment planning of rectum, anal canal, cervix and endometrial malignancies.

**Patients and methods:**

Forty patients receiving radiation treatment for a range of pelvic malignancies, were separated into male (n = 20) and female (n = 20) cohorts for the creation of gender specific atlases. A multi-atlas local weighted voting method was used to generate a sCT from a T1-weighted VIBE DIXON MRI sequence. The original treatment plans were copied from the CT scan to the corresponding sCT for dosimetric validation.

**Results:**

The median percentage dose difference between the treatment plan on the CT and sCT at the ICRU reference point for the male cohort was − 0.4% (IQR of 0 to − 0.6), and − 0.3% (IQR of 0 to − 0.6) for the female cohort. The mean gamma agreement for both cohorts was > 99% for criteria of 3%/2 mm and 2%/2 mm. With dose criteria of 1%/1 mm, the pass rate was higher for the male cohort at 96.3% than the female cohort at 93.4%. MRI to sCT anatomical agreement for bone and body delineated contours was assessed, with a resulting Dice score of 0.91 ± 0.2 (mean ± 1 SD) and 0.97 ± 0.0 for the male cohort respectively; and 0.96 ± 0.0 and 0.98 ± 0.0 for the female cohort respectively. The mean absolute error in Hounsfield units (HUs) within the entire body for the male and female cohorts was 59.1 HU ± 7.2 HU and 53.3 HU ± 8.9 HU respectively.

**Conclusions:**

A multi-atlas based method for sCT generation can be applied to a standard T1-weighted MRI sequence for male and female pelvic patients. The implications of this study support MRI only planning being applied more broadly for both male and female pelvic sites.

*Trial registration* This trial was registered in the Australian New Zealand Clinical Trials Registry (ANZCTR) (www.anzctr.org.au) on 04/10/2017. Trial identifier ACTRN12617001406392.

**Supplementary Information:**

The online version contains supplementary material available at 10.1186/s13014-022-02023-4.

## Background

Computed tomography (CT) is the long established imaging modality used for radiation therapy treatment planning. The inherent electron density [derived from grey scale Hounsfield units (HU)] and anatomical data from the CT scan, is used by commercial computer planning systems to model and calculate radiation dose distribution within the patient’s body, using specific calculation algorithms [1]. The limited soft tissue contrast and tumour delineation however, has given rise to the increasing use of Magnetic Resonance Imaging (MRI) in this context [2, 3]. As such, dedicated MRI scanners and MRI-linear accelerator hybrid machines are increasingly being deployed in radiation oncology worldwide.

Diagnostic MRI scans are used as a supplement to CT datasets for radiation treatment planning, however this introduces systematic inaccuracies in the planning process due to positional differences between the scans [4, 5]. The MRI simulator affords the ability to scan patients in the treatment position and potentially leads to a reduction in the registration errors. However due to unavoidable differences in patient positioning between the two scans and inherent registration uncertainties, alignment errors are still present. These errors are estimated to be in the order of 2–4 mm for pelvic MRI to CT registrations [6, 7]. This has resulted in increased research to incorporate MRI into radiotherapy planning, using MRI as primary imaging set rather than supplement.

The treatment planning system (TPS) uses electron density, resulting from the photoelectric effect and Compton scattering, to calculate the dosimetry. Unlike CT, the greyscale units of the MRI image do not correlate with the electron density of tissues, therefore the treatment planning system (TPS) is unable to accurately model the dose deposition on a MRI scan. Researchers have developed various methods to create a synthetic CT scan (sCT) from the MRI scan in order to estimate the electron densities of structures, allowing for dose calculations [5]. There has been an increasing focus in machine learning methods of sCT creation, however, current applications in the clinic have relied on atlas based methods [8–12]; this approach has matured and has been validated across multiple sites as compared to machine learning methods.

The atlas based approach of generating sCT, which has been successfully translated to the clinic, involves the creation of an atlas of matching CT and MRI pairs. The MRI scans in the atlas are deformed to the new MRI and the deformation vectors are then applied to the corresponding CT pairs. This was initially performed using a single (average) scan pair atlas [13]. Later Dowling et al. [10] and Arabi et al. [14] further improved on atlas-based sCT generation by presenting a hybrid approach in which a library of CT-MRI pairs is used, combined with local weighting of atlas patch values to create the sCT scan.

Prostate cancer has been the focus of the MRI planning research due to its prevalence, small target, and the lack of complex anatomical interfaces [10, 15]. The treatment volumes for colorectal and gynaecological cancers are much more multifarious, traversing a more variable body contour and bony anatomy than prostate treatments. Rectum, anal canal and gynaecological treatments routinely involve treating the gross tumour volume, surrounding tissue deemed to be at high risk of tumour spread, the disease positive nodes and the surrounding local nodal volumes to different radiation therapy prescriptions [16–19]. Given the treatment volumes for these patients are comparatively larger than prostate patients, there is a need to create a new atlas set for these patients, as well as the requirement for gender specific atlases. There has been limited work in the literature on sCT creation for larger pelvic treatment sites, with small groups of patient numbers and no consideration of the differences in male and female pelvic anatomy [9, 14, 20–22].

This work investigated the application of a hybrid multi-atlas approach for sCT creation for male and female full pelvis treatments using forty patient datasets acquired prospectively. Mean error and mean absolute error in HU, volume comparisons and the Dice Similarity Co-efficient (DSC) were used to assess the anatomical accuracy while percentage dose difference at a reference point, gamma dose comparison and dose volume histogram analysis for relevant structures was used to assess dosimetric accuracy of the sCT generation.

## Materials and methods

### Patient data collection

Ethics approval for the study was obtained through the local health district human research ethics committee (ref:17/06/21/3.02). Forty-one patients receiving radiation treatment for histologically confirmed malignancy of either the rectum, anal canal, cervix or endometrium, gave informed consent to participate in the trial. One patient was excluded after insufficient coverage of the MRI scan due to user error. The remaining forty participants were separated into male (n = 20) and female (n = 20) cohorts for the creation of gender specific atlases.

CT scans were acquired on a SOMATOM Confidence CT scanner (Siemens Healthineers; Erlangen, Germany) at 120 kV with 2.0 mm slice thickness. Patients were positioned supine, legs flat, using a CIVCO vac-lok bag (CIVCO Medical Instruments; Iowa, USA) under their legs. All patients were scanned with a full bladder and empty rectum, and oral or intravenous contrast was administered at the radiation oncologist discretion. Scan range included the whole lumbar spine superiorly, to mid femur inferiorly. Three positioning tattoos were used to aid in patient setup for treatment alignment.

**Fig. 1:**
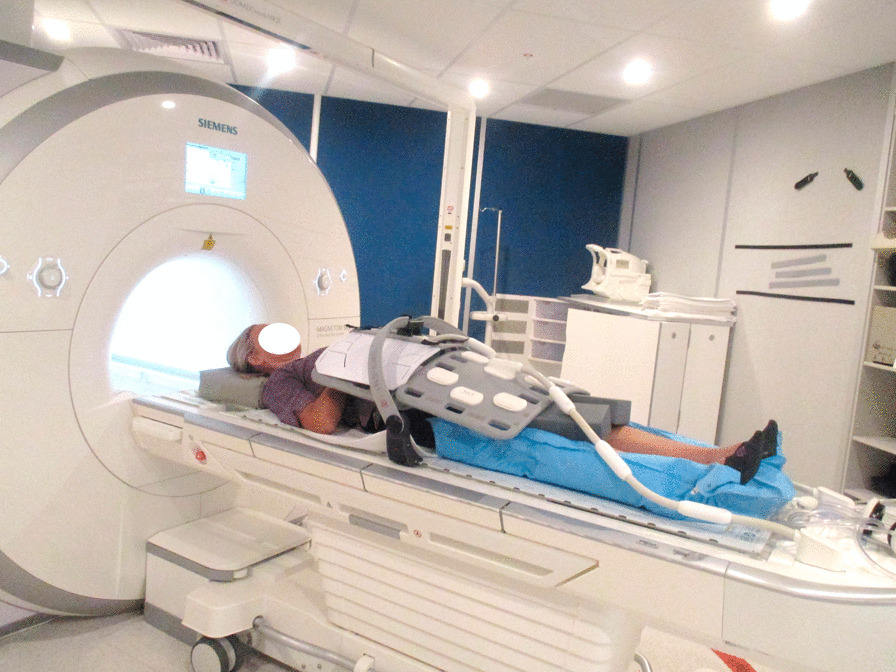
Patient positioning for pelvic MRI using 2 × 18 channel body coils with MRI coil bridge and sponges

MRI scans were performed following the planning CT scan (mean 17.6 ± 13.0 (1 SD) minutes between scans), on a MAGNETOM Skyra 3T MRI scanner (Siemens Healthineers; Erlangen, Germany), to ensure similar bowel and bladder filling. The MRI scanner was equipped with a Qfix flat couch (Qfix; Pennsylvania USA) and DORADOnova MR 3T external laser bridge (LAP; Luneburg, Germany). Patients were positioned by a radiation therapist and a MRI radiographer, using their custom vac-lok bag. Patients were aligned using the positional tattoos and the external laser bridge. A 32 channel spine coil was utilised under the flat couch top and two 18 channel body coils were used over the pelvic region. To avoid compression of the external body contour, one body coil was positioned in a Qfix INSIGHT MR Body coil holder and placed over the superior portion of the field, while the second coil was positioned with the superior edge on the inferior edge of the coil bridge and the inferior edge on sponges (Fig. 1).

For sCT generation, an additional T1 VIBE DIXON sequence was added to the patient’s scanning protocol of a small field of view T2 weighted sequence, and included the entire lumbar spine to mid femur, similar to the field of view of the CT. VIBE is a volumetric imaging technique, which is a fast 3D gradient-echo sequence, producing a T1-weighted image. The T1 VIBE DIXON sequence parameters are outlined in Table 1. To minimise magnetic field related distortion in the MRI sequence for sCT creation:

1. Vendor supplied 3D distortion correction software was applied,
2. The scan was acquired in two stages in the coronal plane. Inline composing was automatically performed using Siemens adaptive algorithm, with 48 mm of overlap,
3. A high receive bandwidth of 1200 Hz/pixel was used to reduce the fat water shift to a sub pixel level (0.3 pixel).

**Table 1:** MRI acquisition parameters

Parameter	T1 VIBE DIXON
Scan type	VIBE DIXON
TE (ms)	1.23/2.46
TR (ms)	4.19
Flip angle	9°
FOV (mm)	256 * 499
Slice thickness (mm)	1.6
Base resolution	160
Acquisition plane	Coronal
Phase direction	R > L
Bandwidth (Hz/px)	1200
Fat–water shift (px)	0.3
Distortion correction	3D
Acquisition stages	2
Overlap (mm)	48
Composing	Inline

The T1 VIBE DIXON scan was acquired in the coronal plane, with an isotropic voxel size of 1.6 mm, as inline composing of an axial acquisition resulted in uneven slice thickness and missing slices at the overlap junction. The Siemens adaptive algorithm uses elastic matching to correct for distortion caused by magnetic field inhomogeneity [23]. The phase encoding direction ran right to left and was extended to 195% of the read field of view, to allow for patient’s hips to be included laterally. The composed scans were then reconstructed axially for sCT creation and imported into the TPS.

Treatment planning was performed as per department protocol on the CT scan using the Eclipse TPS (version 15.6; Varian Medical Systems). Three patients in the male cohort were planned as 6-MV, 7–9 field sliding-window Intensity Modulated Radiation Therapy (IMRT) while all other patients were planned as 6-MV, 2–3 arc Volumetric Modulated Arc Therapy (VMAT).

### sCT creation

A leave-one-out cross validation approach was used to generate sCTs for each of the male and female groups (i.e. 19 patients were used to generate a sCT for each target patient MRI).

The sCT generation method was similar to that used in Dowling et al. [10] with some modifications to account for the larger field of view and female anatomy. All MRIs were pre-processed with N4 bias field correction [24] with background masked to 0. All CT scans had their background masked to − 1000. Bone and bladder structures were contoured on the CT and MRI by a radiation therapist. For atlas generation, the CT was registered to the matching MRI using structure guided registration (using binary labels based on the bone and bladder contours) for both rigid and non-rigid registration using custom code written in simpleITK [25]. For converting each target MRI to sCT, the MRI body contour was required to help guide an initial rigid registration from each of the 19 atlas cases due to the comparatively larger superior-inferior coverage of the data sets. This was followed by deformable registration. The custom initial registration was written using the SimpleITK (https://simpleitk.org/) library and registered distance maps (SimpleITK SignedMaurerDistanceMapImageFilter) from the combined binary labels from the bladder and bones from each modality. These distance maps were initially registered using a rigid registration (6 DoF, metric = MSE), followed by a Fast Symmetric Forces Demons Registration (standard deviation = 1). The transform and deformation fields from these steps were then applied to initial moving CT image to initialize the multi-modal registration. Finally the initial CT-MR registration results was refined with a final step of non-rigid registration using NiftyReg (http://cmictig.cs.ucl.ac.uk/wiki/index.php/NiftyReg) reg_f3d with default parameters (free form deformation, multiscale scale approach, metric = normalized mutual information).

Each MRI to MRI registration was performed initially using the body masks only using reg-aladin from NiftyReg with default parameters apart from -rigOnly (6 degrees of freedom) and -rmask and -fmask with the respective body masks. Following this step, the moving MR image was propagated using the same transform and then deformably registered to the target MRI (using the ITK diffeomorphic demons registration implementation (3 standard deviations, 3 multi-resolution levels).

The co-registered CT scans were propagated using the same deformation fields to the target MRI and then local weighted voting was applied to generate the final sCT volume (using a radius of 2 voxels and a gain of 1). The radius parameter defined the size of the patches used in the atlas-based local weighted voting process: the radius is the offset from the centre voxel. For example, in 3D, a radius of 2 results in a 5 × 5 × 5 patch of voxels. The gain parameter was used to increase sensitivity with the similarity measure between patches (increasing the gain can help differentiate patches with very similar intensity values). The computed weighted similarity between patches in the registered MRI to the target MRI were used to combine the patches in the same location, from co-registered and propagated CT-MR scans in the atlas dataset.

### sCT validation

The T1 VIBE DIXON MRI and sCT were imported into the TPS. For each subject, the sCT was co-registered to the MRI and the CT using rigid registration, with a registration boundary of the top of L2 to the greater trochanter. Co-registration of the sCT and MRI was required, as the frame of reference information was stripped from the data set during sCT generation. Due to a disparity in location of bowel gas on the CT and MRI scans, bowel gas in the proximity of the treatment region was overridden to average surrounding tissue HU value on both the CT and sCT for ten patients. The body was contoured using image thresholding on the CT and sCT and used as the calculation volume for the corresponding data set. Two patients had a large discrepancy in body contour of > 4 cm in the lateral posterior region, between the CT and MRI, due to tensing of the gluteal muscles in CT. This region of discrepancy in patient positioning was removed from the sCT calculation volume for dosimetric analysis alone, so as to not affect the results. An in-house HU to electron density curve (Siemens BR38 kernel) was applied to the CT and sCT. The CT based treatment plan, International Commission of Radiation Units and Measurements (ICRU) reference point and structure set were copied from the original CT to the sCT [26]. The structures were copied using the rigid registration between the CT and sCT. The treatment plan was then re-calculated with identical monitor unit values.

Dosimetric accuracy was assessed using the CT based plan as the gold-standard. The dose difference at ICRU reference point and dose volume histogram (DVH) analysis for relevant planning target volume (PTV) and organ at risk (OAR) structures were assessed. The relevant DVH parameters used for these structures were as per standard guidelines for each treatment site (see Additional file 1 for greater detail on DVH parameters assessed) [16–19]. Several DVH parameters were evaluated for each structure, the average dose difference for each structure is a combined average of each of these parameters per structure. The percentage dose difference was calculated by the formula (D_sCT_ − D_CT_)/D_CT_ * 100%. Statistical significance of the dose difference at ICRU reference point was determined using a Wilcoxon Signed-Rank Test with a significance level of 0.05. Three-dimensional gamma analysis was used to evaluate the dose impact of the sCT on the treatment plan across the entire treatment volume. 3D gamma analysis was performed using an in-house MATLAB code (MATLAB; MathWorks), using a dose-difference (%) and distance to agreement (mm) criteria of 3%/2 mm, 2%/2 mm, and 1%/1 mm. An erosion of 15 mm of the body perimeter was applied to exclude failures which occurred at skin edge due to small unavoidable differences in body contour between data sets, and a 10% low dose threshold was applied.

Hounsfield Unit accuracy was assessed using mean error (ME) and mean absolute error (MAE) in the entire body, bone regions and soft tissue regions for each cohort, to assess the accuracy of the atlas-based sCT model. For ME and MAE calculations, the superior and inferior 3 cm of the sCT data sets was excluded to avoid regions of image degradation due to differences in scan coverage in the atlas sets, and the density override of the bowel gas was not applied for these calculations. Due to differences in the body outline between the sCT and CT, the body MAE and ME calculations were performed within the MRI body contour between the registered CT-MRI and the sCT. To assess anatomical accuracy a Dice similarity coefficient (DSC = 2[A ∩ B]/[A + B]) of body and bone regions between the MRI and sCT was calculated, and volume comparisons of bone and body structures were performed between the sCT, CT.

## Results

Detailed patient demographics are outlined in Table 2. Of the 40 patients recruited to the trial, two patients in the male cohort had previous rectal resections, and six patients in the female cohort had previous hysterectomies. Four patients in the male cohort and eleven patients in the female cohort received iodine based oral contrast, while one patient in the female cohort received iodine based IV contrast.

**Table 2:** Patient demographics

	Cohort size	Age range	BMI range (kg/m^2^)	Relevant surgical history	Primary treatment site	Staging range	Prescribed dose
Male cohort	20	49–88 (mean = 65)	20.5–33.6 (mean = 25.5)	Hernia repairs (n = 3) Rectal resections (n = 2) Appendectomy (n = 1)	Rectum (n = 20)	T1N0–T4N1	60 Gy/30fx (n = 1) 50.4 Gy/28fx (n = 1) 50 Gy/25fx (n = 18)
Female cohort	20	41–85 (mean = 61)	18.0–36.9 (mean = 26.2)	Hysterectomy (n = 6) Common iliac stent (n = 1) Caesarean (n = 1) Hernia Repair (n = 2) Appendectomy (n = 3)	Rectum (n = 4)	T3N0–T3N2	50 Gy/25fx (n = 4)
					Anal Canal (n = 4)	T1N0–T3N1	54 Gy/30fx (n = 2) 50.4 Gy/28fx (n = 1) 50 Gy/25fx (n = 1)
					Cervix (n = 8)	IIA–IIB	55 Gy/25fx (n = 1) 50 Gy/25fx (n = 4) 45 Gy/25fx (n = 3)
					Endometrium (n = 4)	IIIA–IIIC	54 Gy/30fx (n = 1) 50 Gy/25fx (n = 1) 45 Gy/25fx (n = 2)

### Dose impact

**Fig. 2:**
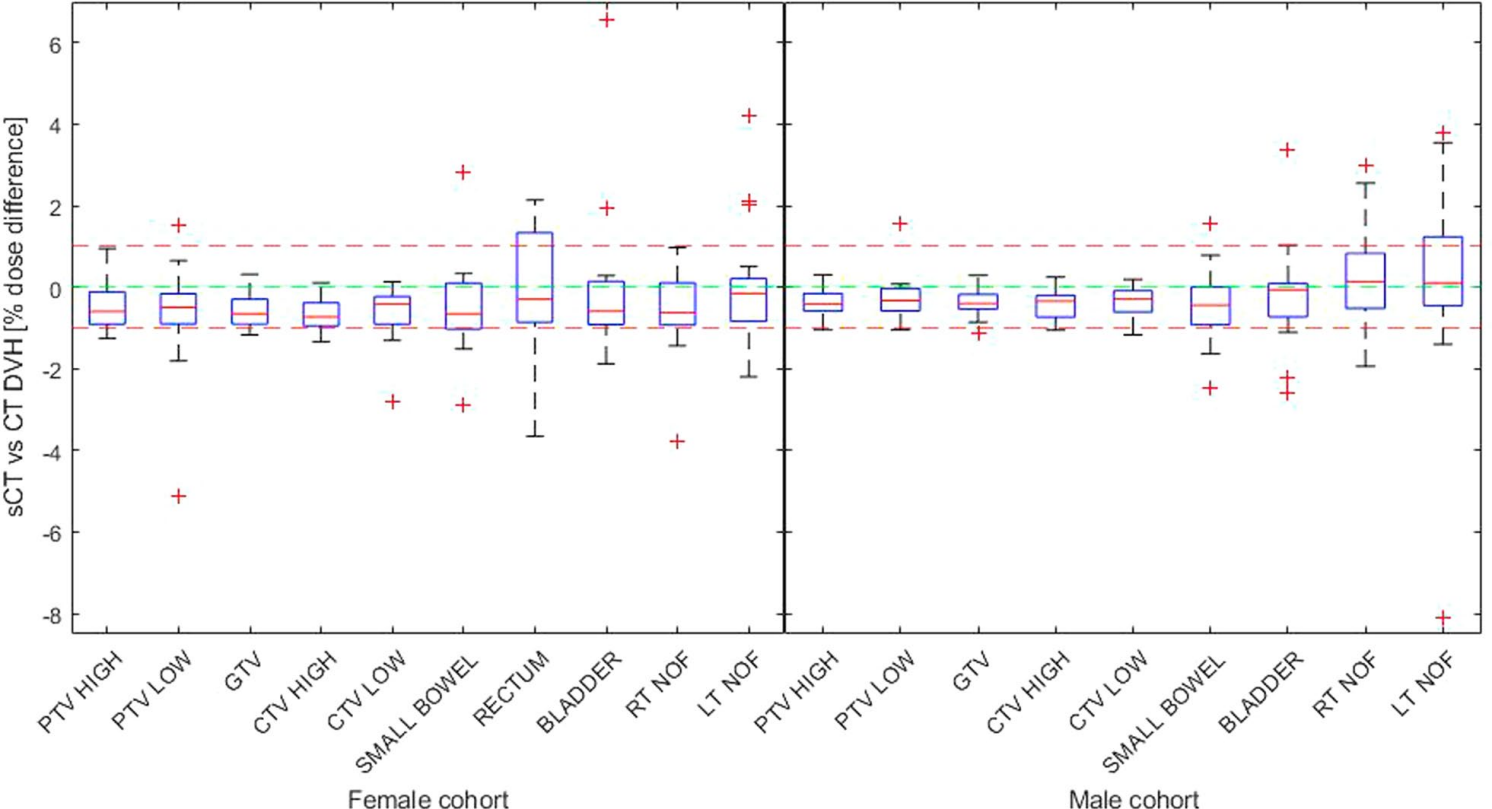
Percentage DVH dose difference by structure (each structure parameters combined) for male and female cohorts. PTV High = Planning target volume higher prescribed dose, PTV Low = Planning target volume lower prescribed dose, GTV = Gross tumour volume, CTV High = Clinical target volume higher prescribed dose, CTV Low = Clinical target volume lower prescribed dose, RT NOF = Right neck of femur, LT NOF = Left neck of femur

There were no statistically significant dose difference at reference point between the treatment plan calculated on the CT and sCT for the female cohort, while there was a statistically significant difference for the male. The median percentage dose difference at the ICRU reference point was − 0.4% (interquartile range (IQR) of 0.0 to − 0.6, *p* = 0.01) in the male cohort, and − 0.3% (IQR of 0.0 to − 0.6, *p* = 0.10) in the female cohort. The median DVH percentage dose difference of all DVH parameters combined was − 0.2% (IQR of 0.2 to − 0.7, *p* =  < 0.05) for the male cohort and − 0.4% (IQR of − 0.1 to − 0.9, *p* =  < 0.05) for the female cohort (Fig. 2). See Additional file 2 for detailed separated DVH dose difference results.

**Fig. 3:**
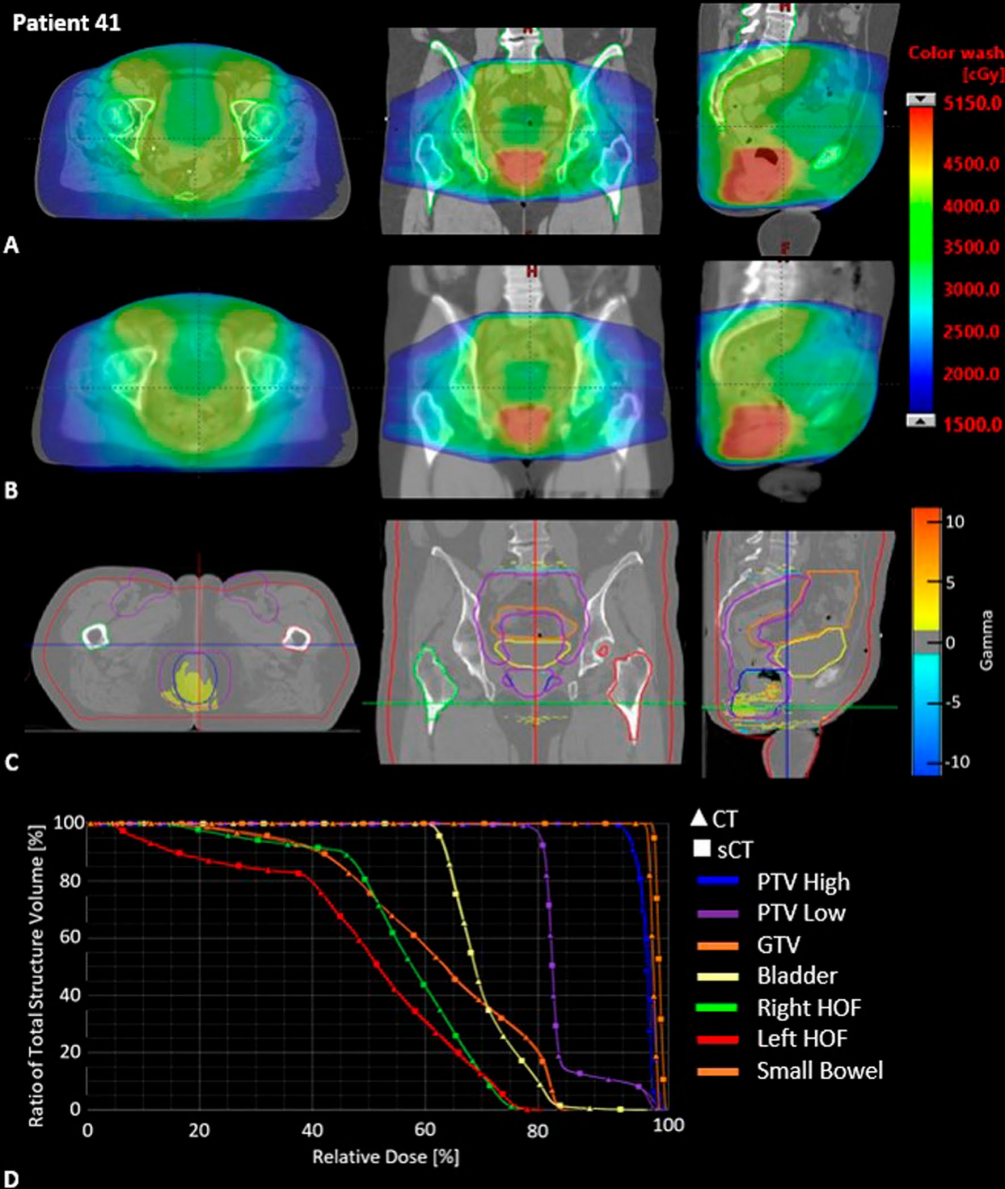
Results for patient 41 (worst performing gamma). Row **A** Original patient CT scan with dose overlayed. Row **B** sCT with dose overlayed. Row **C** CT scan and critical structure outlines with Gamma map overlayed (1%/1 mm) colour wash showing regions which do not meet the gamma pass rate (values between 1 and − 1 not displayed). Row **D** Dose volume histogram results

The 3D gamma results with criteria of 3%/2 mm for all patients were within the American Association of Physics in Medicine (AAPM) TG218 report guidelines of > 95% (Table 3) [27]. Figure 3 visually represents the 3D gamma with criteria of 1%/1 mm for the worst performing gamma (patient 41). The area of colour wash in row C represents the regions which do not meet the gamma criteria, this is occurring in a high dose region close to a steep dose drop off and penumbra region at the lower aspect of the field, mostly in the inferior aspect of the PTV high region. The DVH in row D shows similarity in dose to structures between the CT plan and sCT plan overlayed.

**Table 3:** 3D Gamma analysis results for male cohort (n = 20) and female cohort (n = 20) (mean ± 1 SD)

	3%/2 mm		2%/2 mm		1%/1 mm	
	Pass rate (%)	Av Gamma	Pass rate (%)	Av Gamma	Pass rate (%)	Av Gamma
Male cohort	99.8 $\pm$ 0.2	0.10 $\pm$ 0.03	99.7 $\pm$ 0.3	0.15 $\pm$ 0.04	96.3 $\pm$ 3.1	0.31 $\pm$ 0.09
Range	100.0–99.3	0.07–0.18	100.0–99.0	0.11–0.25	99.2–88.7	0.21–0.52
Female cohort	99.8 $\pm$ 0.3	0.13 $\pm$ 0.04	99.7 $\pm$ 0.4	0.19 $\pm$ 0.05	93.4 $\pm$ 5.2	0.38 $\pm$ 0.12
Range	100.0–99.1	0.08–0.19	100.0–98.8	0.11–0.28	99.1–81.0	0.23–0.57

### Anatomical accuracy

Agreement in the average Dice similarity co-efficient for the bone and body regions, between the MRI and sCT is shown in Table 4. The volume comparisons between the sCT and CT resulted in a − 2.1% and − 1.4% whole body volume difference and a − 3.1% and − 4.1% bone volume difference for the male and female cohort respectively.

**Table 4:** DSC and volume comparison for body and bone structures. Mean absolute error and mean error in HU (mean ± 1 SD)

	sCT versus MRI		sCT versus CT					
	DSC		Vol. difference (%)		MAE (HU)		ME (HU)	
	Male	Female	Male	Female	Male	Female	Male	Female
Body	0.97 ± 0.0	0.98 ± 0.0	− 2.1 ± 2.0	− 1.4 ± 1.8	59.1 ± 7.2	53.3 ± 8.9	− 18.8 ± 11.0	− 16.7 ± 14.3
Bone	0.91 ± 0.2	0.96 ± 0.0	− 3.1 ± 2.9	− 4.1 ± 2.1	166.7 ± 19.8	171.2 ± 26.6	− 118.5 ± 33.6	− 129.1 ± 34.1

*CT* computed tomography, *sCT* synthetic CT, *MRI* magnetic resonance imaging, *DSC* dice similarity co-efficient, *MAE* mean absolute error, *ME* mean error, *HU* hounsfield unit

**Fig. 4:**
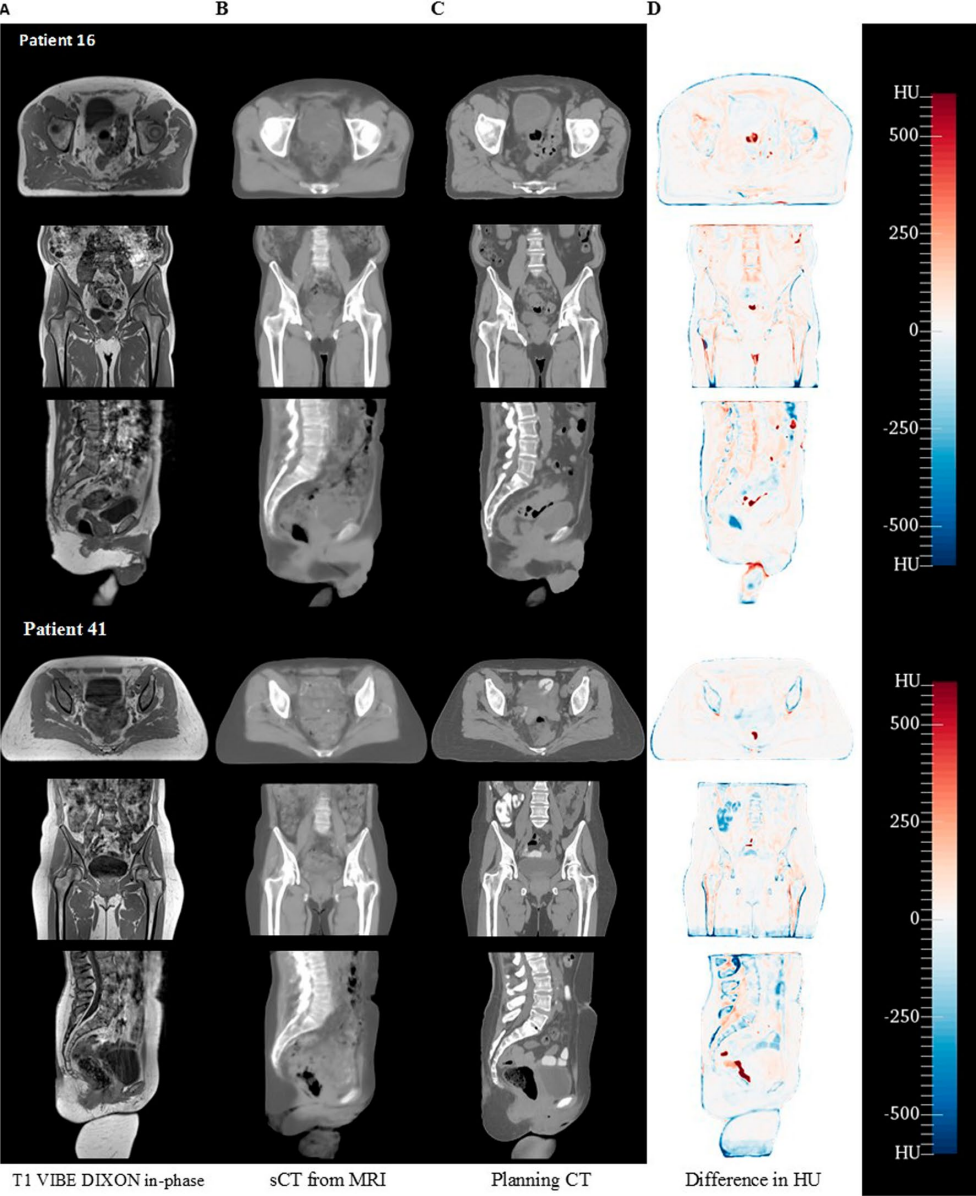
HU difference results for the best and worst performing gamma. Column **A** Original T1 VIBE DIXON in-phase MRI from patient 16 (best gamma) and patient 41 (worst gamma); **B** sCT generated from MRI; **C** planning CT scan; **D** HU difference between sCT and CT

The mean absolute error in HU of the body between the CT and sCT was 59.1 $\pm$ 7.2 for the male cohort and 53.3 $\pm$ 8.9 for the female cohort (Fig. 4). The mean absolute error in the bone regions for the male and female cohort was 166.7 $\pm$ 19.8 and 171.2 $\pm$ 26.6 respectively.

## Discussion

The results presented in this article are comparable to previous studies on MRI only planning for pelvic treatments. Dowling et al. [13] originally applied a single atlas approach to sCT generation for prostate cancer treatments, with a dose difference at ICRU reference point of 1.5%. The same group furthered this work by using a multi-atlas hybrid approach for sCT generation on a similar cohort to the original study, and the results greatly improved [10]. This study applies Dowling et al.’s [10] multi-atlas hybrid approach to greater pelvic regions and male and female cohorts. The reported MAE and ME in HU of the body contours was of greater accuracy in Dowling et al.’s [10] study than both cohorts in this study, however the dose difference at ICRU reference point of − 0.3% $\pm$ 0.8%, DVH dose difference < 0.5% and gamma results > 95.0% at 1%/1 mm were very similar to this study. While Dowling et al. previously used a T2 weighted image, this study utilised a T1-weighted VIBE DIXON imaging technique, which is increasingly favoured in recent MRI planning studies due to; better anatomical definition of T1-weighting; typically shorter echo times (TE), to include tissues with short TE properties; reduced scan time for a larger field of view; as well as the benefit of having an in-phase, out-of- phase, fat-weighted and water-weighted image sets.

Studies which have investigated atlas-based MRI only planning for larger pelvic treatment sites have had relatively low patient numbers compared to this study, and did not focus on optimising sCT methods for separate genders. Arabi et al. [14] utilised an atlas based sCT generation with local weighted voting using a T1 VIBE DIXON MRI on 12 patients with rectal cancer (2 female, 10 male). Using a single atlas for both genders, Arabi et al. [14] achieved a bone Dice of 0.89 and the OAR dose difference mean was less than 0.9%, with the gamma criteria of 2%/2 mm at 99.86% ± 0.27%, and 1%/1 mm at 97.67% ± 3.60%.

Other studies investigating MRI planning for rectal and gynaecological treatments have focused on a tissue class segmentation approach. Maspero et al. [21], and Kemppainen et al. [20] both utilised a commercially available product by Philips healthcare, MRCAT. Both studies found similar results when applying this method to male and female cohorts. Maspero et al. applied MRCAT to fifteen male and five female patients with rectal malignancies, the resulting gamma pass rate at 2%/2 mm was 94.7% $\pm$ 1.7%, and on average a mean increase of 0.3% to the dose to target [21]. Kemppainen et al. [20] found better agreement using MRCAT software for rectal and gynaecological treatment, with median relative dose difference to PTV less than 0.8% and the median relative dose difference to OARs was less than 1.2%. A higher gamma pass rate was found with a criteria of 2%/2 mm, with the rectum cohort and gynaecological cohort being 99.3% and 99.2% respectively.

Liu et al. [9] applied a shape model for bone with a modified probabilistic tissue classification using shape classification to T1 VIBE DIXON images of 10 female patients with pelvic malignancies, resulting in a maximal mean dose difference of 0.3 Gy (0.5%). Wang et al. [22] applied a bone mask and tissue class segmentation to VIBE DIXON images of 11 patients with rectal cancer (9 female, 2 male). The reported median dose difference in the target volume was 0.3% and the median gamma pass rate was higher than 99% for 2%/2 mm criteria.

In this study we reported a high level of agreement in dosimetry and gamma pass rate for both male and female cohorts, which compares equivalently to the above mentioned MRI planning studies for the same body region. The sCT created from both atlases performed equally well. The anatomical accuracy measured with the DSC between the MRI and sCT was high, with the bony anatomy showing the greatest variation in scores. The cases showing a lower DSC score for bone regions could be due to the difficulty in identifying and contouring bone regions on MRI, introducing some inaccuracies, as well as the ability of the atlas to account for greater variations in anatomy from the atlas sets. This factor could be resolved with a greater number of data sets within the atlas to represent a greater variety of anatomy differences. In this study it is difficult to isolate the bony disagreement as the reason for lower dosimetry agreement between the CT and sCT due to several compounding factors affecting dosimetry. Further work could be done to isolate the bony anatomy to determine the effect it has on dosimetry alone.

Although an attempt was made to account for unavoidable differences between the CT and MRI, such as adjusting the body contour for set up variations; other variations which may affect results, such as the presence of oral contrast being greater in the female cohort (55%) than the male cohort (20%) and previous surgeries can affect results. We did not attempt to control these, so as to mirror routine clinical presentations. Although it is conceivable that this could lead to different pass rates, however, most differences were found at the field edges bearing the penumbra regions, which is a known area of failure with CT based planning. An attempt was also made to control the difference in bowel gas placement between the CT and MRI by performing a density override of bowel gas on both the CT and sCT for dosimetric analysis alone. Importance was not placed on the sCT ability to accurately recreate bowel gas from the MRI scan in this study, due to the variability in bowel gas placement between simulation and treatment day to day. Due to this, it is practice in our department to override bowel gas on simulation scans for treatment planning to account for this day to day variability, and therefore this practice was mirrored in the analysis of the sCT.

In this study, a stitched T1 VIBE DIXON sequence was utilised for sCT generation. To minimise distortion effects associated with the magnetic field inhomogeneity and patient susceptibility a read-out bandwidth of 1200 Hz/Px was selected, with a fat–water shift of 0.3px; below 1 mm. Furthermore a two stage acquisition with overlapping stages to scan at isocentre and 3D distortion correction was applied to reduce gradient non-linearity distortion effects. On phantom testing, geometric distortion, non-linear gradient fields and distortion relating to bandwidth was measured in the order of 1–2 mm for the field of view used in this study. As the geometrical uncertainties in the MRI translate to the sCT, this analysis of the sCT also addresses the effect this inherent distortion has when carried through to treatment planning.

All of factors mentioned were found to minimally affect the DVH dose impact to the target volumes and OARs and be within acceptable guidelines. We found this method to be relatively simple to implement as part of an MRI planning workflow, using a standard MRI sequence and with a high level of DSC agreement in the bony contours which is also important for image guided radiation therapy treatment.

## Conclusion

This study has shown that a multi-atlas based method for sCT created from routinely employed MRI sequences can be used for definitive pelvic radiotherapy planning for male and female patients. The implications of this study means that MRI planning can be applied more broadly to male and female cohorts and more treatment regions in the pelvis, therefore greatly expanding the scope of MRI only planning.

## Supplementary Information

**Additional file 1.** DVH data collection table. Organ at risk and target volume DVH parameters assessed by treatment site.

**Additional file 2.** Detailed DVH results table. Organ at risk and target volume DVH dose difference separated by individual parameters for male and female cohorts. *Note*: Some structure parameters are not included as sample size is too small for individual analysis.

## Data Availability

The datasets used during the study are available from the corresponding author on reasonable request.

## Abbreviations

CT: Computed tomography
DSC: Dice similarity coefficient
DVH: Dose volume histogram
HU: Hounsfield units
ICRU: International Commission of Radiation Units and Measurements
IQR: Interquartile range
IMRT: Intensity modulated radiation therapy
MRI: Magnetic resonance imaging
MAE: Mean absolute error
ME: Mean error
OAR: Organ at risk
PTV: Planning target volume
sCT: Synthetic CT
TPS: Treatment planning system
VIBE: Volumetric interpolated breath-hold examination
VMAT: Volumetric modulated arc therapy
