# Supplementary material for: Validation of an MRI-only planning workflow for definitive pelvic radiotherapy
Source: Radiat Oncol. 2022 Mar 18;17:55. doi: 10.1186/s13014-022-02023-4 (PMC8932060; doi:10.1186/s13014-022-02023-4)
Supplement: Supplementary file 1 — Additional file 1. DVH data collection table. Organ at risk and target volume DVH parameters assessed by treatment site. [file 13014_2022_2023_MOESM1_ESM.pdf]

ADDITIONAL FILE 1:

DVH data collection table: Organ at risk and target volume DVH parameters assessed by treatment site

Primary Disease Site	Prescribed Dose	Organ at Risk	Dose parameters assessed
**Rectum (n=24)**	60Gy/30fx (n=1)<br>50.4Gy/28fx (n=1)<br>50Gy/25fx (n=22)	GTV	D95%
		CTV high	D95%
		CTV low	D95%
		PTV high	D2%<br>D95%
		PTV low	D2%<br>D95%
		Small Bowel	D2%<br>D20%
		Bladder	D20%<br>D40%
		Right Neck of Femur	D40%<br>D25%
		Left Neck of Femur	D40%<br>D25%
**Anal Canal (n=4)**	54Gy/30fx (n=2)<br>50.4Gy/28fx (n=1)<br>50Gy/25fx (n=1)	GTV	D95%
		CTV high	D95%
		CTV low	D95%
		PTV boost (n=1)	D2%<br>D95%
		PTV high	D2%<br>D95%
		PTV low	D2%<br>D95%

		Small Bowel	D2%
			D20%
		Bladder	D20%
			D50%
		Right Neck of Femur	D50%
		Left Neck of Femur	D50%
		Genitalia	V50%
**Cervix (n=8)**	55Gy/25fx (n=1)	CTV high	D95%
	50Gy/25fx (n=4)	CTV low	D95%
	45Gy/25fx (n=3)	PTV boost (n=1)	D95%
		PTV high	D2%
			D10%
			D95%
		PTV low	D2%
			D95%
		Small Bowel	D2%
			D20% (n= 3)
			D25% (n= 5)
		Bladder	D20%
			D35%
		Right Neck of Femur	D25%
		Left Neck of Femur	D25%
		Rectum	D20%
			D60%
**Endometrium (n=4)**	54Gy/30fx (n=1)	CTV high	D95%
	50Gy/25fx (n=1)	CTV low	D95%

	45Gy/25fx (n=2)	PTV high	D2%
			D10%
			D95%
		PTV low	D2%
			D95%
		Small Bowel	D2%
			D25%
		Bladder	D20%
			D35%
		Right Neck of Femur	D25%
		Left Neck of Femur	D25%
		Rectum	D20%
			D60%

PTV High = Planning target volume higher prescribed dose, PTV High Boost = Planning target volume dose escalation region, PTV Low = Planning target volume lower prescribed dose, GTV= Gross tumour volume, CTV High = Clinical target
