# Supplementary material for: Validation of an MRI-only planning workflow for definitive pelvic radiotherapy
Source: Radiat Oncol. 2022 Mar 18;17:55. doi: 10.1186/s13014-022-02023-4 (PMC8932060; doi:10.1186/s13014-022-02023-4)
Supplement: Supplementary file 2 — Additional file 2. Detailed DVH results table. Organ at risk and target volume DVH dose difference separated by individual parameters for male and female cohorts. Note: Some structure parameters are not included as sample size is too small for individual analysis. [file 13014_2022_2023_MOESM2_ESM.pdf]

ADDITIONAL FILE 2:

Detailed DVH results table: Organ at risk and target volume DVH percentage dose difference separated by individual parameters for male and female cohorts. Note: Some structure parameters are not included as sample size is too small for individual analysis.

OAR DVH parameter	Cohort	Median (% dose difference)	Inter-quartile range	Wilcoxon signed-rank test p-value
**PTV High D10%**	Female	-0.6	-0.4 to -1.0	<0.05
**PTV High D2%**	Female	-0.6	-0.4 to -1.0	<0.05
	Male	-0.5	-2.1 to -6.3	<0.05
**PTV High D95%**	Female	-0.6	-0.1 to -0.8	0.07
	Male	-0.3	-0.1 to -0.6	<0.05
**PTV Low D2%**	Female	-0.6	-0.3 to -1.1	<0.05
	Male	-0.4	-0.2 to -0.6	<0.05
**PTV Low D95%**	Female	-0.4	0.1 to -0.9	0.61
	Male	-0.3	0.1 to -0.6	<0.05
**GTV D95%**	Female	-0.8	0.1 to -1.0	<0.05
	Male	-0.4	-0.2 to -0.6	<0.05
**CTV High D95%**	Female	-0.8	-0.4 to -1.0	<0.05
	Male	-0.4	-0.2 to -0.8	<0.05
**CTV Low D95%**	Female	-0.4	-0.2 to -0.9	<0.05
	Male	-0.3	-0.1 to -0.6	<0.05
**Small Bowel D2%**	Female	-0.3	0.2 to -0.9	0.06
	Male	-0.4	0.1 to -0.8	0.09
**Small Bowel D20%**	Female	0.0	0.3 to -1.2	0.72
	Male	-0.5	0.0 to -1.1	<0.05
**Small Bowel D25%**	Female	-0.6	0.2 to -1.4	<0.05
**Bladder D20%**	Female	-0.6	0.2 to -0.9	0.06
	Male	-0.1	0.2 to -0.9	0.19
**Bladder D40%**	Male	-0.1	0.2 to -0.3	0.24
**Bladder D35%**	Female	-0.8	-0.5 to -1.2	<0.05
**RT NOF D25%**	Female	-0.8	0.5 to -1.0	<0.05
	Male	0.0	0.7 to -0.4	0.67
**LT NOF D25%**	Female	-0.1	0.4 to -0.6	0.90
	Male	0.0	1.1 to -0.5	0.58
**Rectum D20%**	Female	-0.4	0.6 to -1.1	0.13
**Rectum D60%**	Female	0.1	0.6 to -0.5	0.87

PTV High = Planning target volume higher prescribed dose, PTV Low = Planning target volume lower prescribed dose, GTV= Gross tumour volume, CTV High = Clinical target volume higher prescribed dose, CTV Low = Clinical target volume lower prescribed dose, RT NOF = Right neck of femur, LT NOF = Left neck of femur
